# Authoritative parent feeding style is associated with better child dietary quality at dinner among low-income minority families

**DOI:** 10.1093/ajcn/nqy142

**Published:** 2018-08-30

**Authors:** Katherine R Arlinghaus, Kirstin Vollrath, Daphne C Hernandez, Shabnam R Momin, Teresia M O'Connor, Thomas G Power, Sheryl O Hughes

**Affiliations:** 1 Department of Health and Human Performance, University of Houston, Houston, TX; 2 Department of HEALTH Research Institute, University of Houston, Houston, TX; 3 USDA–Agricultural Research Service, Children's Nutrition Research Center at Baylor College of Medicine, Houston, TX; 4 Department of Human Development, Washington State University, Pullman, WA

**Keywords:** child dietary quality, direct observation, parent feeding styles, preschoolers, Healthy Eating Index

## Abstract

**Background:**

Parent feeding styles have been linked to child weight status across multiple studies. However, to our knowledge, the link between feeding styles and children's dietary quality, a more proximal outcome, has not been investigated.

**Objective:**

The purpose of this study was to examine the relation between parent feeding styles and dietary quality of Head Start preschoolers’ dinner meals.

**Design:**

The amount of food served and consumed by children was measured by using a standardized digital photography method during 3 in-home dinner observations of low-income minority families in Houston, Texas. Trained dietitians entered food served and consumed into the Nutrient Data System for Research 2009 for nutrient analysis. Overall dietary quality of the food served and consumed at dinner was evaluated by using the Healthy Eating Index 2010 (HEI-2010). Parent feeding style was assessed with the use of the Caregiver's Feeding Style Questionnaire (CFSQ). On the basis of a parent's level of demandingness and responsiveness to his or her child during feeding, the CFSQ categorizes parent feeding into 4 styles: authoritative (high demandingness and high responsiveness), authoritarian (high demandingness and low responsiveness), indulgent (low demandingness and high responsiveness), or uninvolved (low demandingness and low responsiveness).

**Results:**

For the overall sample, the mean ± SD HEI score for dinner served was 44.2 ± 8.4, and the mean ± SD HEI score for dinner consumed was 43.4 ± 7.0. In the fully adjusted model, ANCOVA indicated that the authoritative parent feeding style was associated with significantly higher child dietary quality compared with the authoritarian feeding style (mean ± SEE HEI consumed—authoritative 45.5 ± 0.9; authoritarian: 41.9 ± 0.7; *P* = 0.001).

**Conclusions:**

Parent feeding style contributes to the overall dietary quality of children, and among low-income minority preschoolers an authoritative feeding style was associated with the highest dietary quality of the 4 feeding styles. Interventions to promote feeding practices that contribute to authoritative feeding are needed to improve the dietary quality of preschool children at dinner. This trial was registered at https://clinicaltrials.gov as NCT02696278.

## INTRODUCTION

Parental feeding styles have consistently been linked to child weight status (1–6) and some child eating behaviors (7–10) across studies in low-income families. However, to our knowledge, no study has linked styles of feeding to the overall quality of the child's diet on the basis of a series of dinner meals. Poor dietary quality in young children can lead to adverse health consequences as children age (11). Preschool age is an important developmental period for adopting healthy eating habits because eating behaviors and preferences established during this time are likely to persist through childhood (12) and into adulthood (13).

Parents are direct drivers of children's dietary quality, and consequently have a unique role in the establishment of children's eating environments (14). Originally developed by Baumrind (15) and extended by Maccoby and Martin (16), general parenting styles categorize overall patterns of parenting behavior on the basis of 2 underlying dimensions of demandingness (the extent to which parents show control, maturity demands, and supervision) and responsiveness (the extent to which parents show affective warmth, acceptance, and involvement). On the basis of this work, parent feeding styles were developed to assess an overall attitude and emotional climate specifically with regard to child feeding (2–5). Parent feeding styles are an optimal way to measure feeding because they encompass the behavioral, social, and nutritional aspects of the parent-child feeding dynamic, including the entirety of family eating from meal conceptualization to consumption (2–5). Feeding styles are conceptualized differently from goal-oriented feeding practices. As with general parenting styles, feeding styles are measured along the dimensions of demandingness and responsiveness. Differences in the dimensions of demandingness (persistence during eating episodes) and responsiveness (sensitivity to the child's individual needs during feeding) result in 4 feeding categories: authoritative (high demand and high response), defined as reasonable nutritional demands in conjunction with sensitivity toward the child; authoritarian (high demand and low response), defined as high control with little sensitivity during feeding; indulgent (low demand and high response), defined as high responsivity with little structure around feeding; and uninvolved (low demand and low response), defined as a lack of involvement during feeding (2–5).

Direct comparison of previous studies regarding feeding styles and diet is difficult due to varying child age groups and eating behaviors targeted as outcomes in the study designs (7–10). Importantly, most of these studies relied on parent dietary recall of child consumption instead of direct observation (17), did not assess the dietary quality of the food served compared with what was consumed, and assessed the quality of specific food groups (i.e., fruit, vegetables, whole grains) instead of overall dinner consumption patterns (8–10). It is important to assess the quality of the overall meal because dietary patterns are more indicative of disease risk than individual foods or nutrients alone (18). For example, findings from experimental and longitudinal studies examining the relation between specific foods, such as fruit and vegetables, and child weight status have not been consistent (19). Although investigation into specific food groups would be expected to relate to overall dietary quality, a more comprehensive assessment of dietary quality is likely necessitated to better understand the relation between dietary consumption and health outcomes. Because parent feeding style is a construct used to better understand the relation between parental feeding and child health outcomes, the potential association between overall dietary quality and parent feeding style is important to examine.

The purpose of this study was to compare the overall dietary quality using the Healthy Eating Index (HEI) of Head Start preschoolers’ dinner meals by parent feeding styles. In the general parenting literature, the authoritative parenting style (high demandingness and high responsiveness) has repeatedly been associated with positive child outcomes, including higher academic achievement, fewer high-risk adolescent behaviors, and greater child maturity (20). Specific to health, the general authoritative parenting style has been associated with improved weight status (21) and may serve as a moderator of the relation between feeding practices and child fruit and vegetable consumption (22, 23). Because feeding styles focus on general parenting dimensions in a specific eating context, it is likely that they are more importantly linked to child dietary quality than parenting style.

Therefore, it was hypothesized that the authoritative feeding style (high demandingness and high responsiveness) would be associated with a higher dinner dietary quality than the other 3 parent feeding styles. This study also had an exploratory aim to compare the dietary quality of the dinner meal served with that of the meal consumed overall and within each of the parent feeding styles. It was hypothesized that the dietary quality of the dinner served would be higher than that consumed within all 4 feeding styles.

## METHODS

### Subjects

Caregivers and their preschool children, who were enrolled in 33 Head Start centers in 3 districts in Houston, Texas, were recruited during 2007 and 2008. Approximately 2500 families were approached during recruitment. Of these, 275 families returned the flyer that was handed out to indicate their interest in participating. All caregivers self-identified their ethnicity as either African American or Hispanic. A caregiver was defined as the individual most responsible for food intake of the child outside of the Head Start day. The caregiver was designated as the target parent in this study. Most caregivers were parents, with a small number of grandparents. Subsequently, throughout the article, caregivers will be referred to as parents. Subjects in this study were part of a larger project that examined parent-child interactions at the dinner meal. Details of the primary analysis of this study have previously been published (4), as have details of initial dietary intake analysis (24). The flow of participants is depicted in a diagram in **Supplemental Figure 1**.

### Procedures

Parents were recruited during parent meetings, when their children were dropped off or picked up at Head Start, and through sign-up sheets posted at the centers. Written consent for the parent and his or her preschooler's participation in the study was obtained. Researchers completed 3 in-home observations of participants’ dinner meals. Parents were instructed to proceed as they normally would at a dinner meal. Eighty-five percent of the meals were eaten at dinner tables. In half of the families, the parent and child ate together. In 43% of the observations, other family members were present during the meals. Researchers provided standardized plates, bowls, and cups, and the food served was documented using a digital photography method. Additional helpings were observed and recorded. It was also noted whether the child was served by the parent or if the child served him- or herself. After the meal was completed, researchers measured plate waste to the nearest 0.1 g on a digital scale (Ohaus Model Pro Scout SP601 scale). Details of this procedure have been described elsewhere (22). At the end of each observed meal, parents were financially compensated (totaling $125 over the 3 observations). This study was approved by the Institutional Review Board at Baylor College of Medicine, and was registered at https://clinicaltrials.gov as NCT02696278.

### Measures

#### Meal representativeness and dietary quality

To assess meal representativeness, parents were asked whether the meal was a typical dinner eaten by their family. Detailed dinner menu information, including recipes, preparation, and brand names of foods, was collected from the parent at each observation, and caregivers reported whether or not the meal they ate was a usual meal for them. The amount of food served was estimated by 2 registered dietitians trained in the digital photography method (24). This information was compiled and entered into the Nutrient Data System for Research 2009 (NDSR) for nutrient analysis. Foods were broken down into the food groups and components utilized in the 2010 Dietary Guidelines for Americans (25).

The dietary quality of the child's dinner meal was evaluated using the HEI-2010 (26, 27). The HEI-2010 evaluates adherence to the 2010 Dietary Guidelines for Americans (25) on a scale from 0 to 100, with a score of 100 being the highest score possible. The HEI is a validated measure of dietary quality because it has been shown to be associated with improved health outcomes (28, 29). At the time of this study, the HEI had not yet been updated for the 2015 Dietary Guidelines for Americans. Because there are minimal differences between how the HEI-2010 and HEI-2015 are calculated (30), it is unlikely that HEI-2015 scores would meaningfully differ from the HEI-2010 scores. The food-group breakdown from the NDSR nutrient analysis was used to calculate HEI-2010 component scores, which were summed to calculate a total HEI-2010 score (26). Adequacy components include total fruit, whole fruit, total vegetables, greens and beans, whole grains, dairy, protein foods, seafood and plant protein, and fatty acids. Moderation components, for which higher scores indicate lower consumption, include refined grains, sodium, and empty calories. The HEI-2010 has been designed so that no one food is required to achieve a perfect score. This enables the HEI-2010 to evaluate a variety of meal patterns (vegetarian, vegan, omnivore) and is inclusive of diets typically consumed by a wide range of ethnic and cultural groups (26). Although the HEI-2010 is typically used to evaluate a person's daily intake, because the index considers food relative to its energy content (27) the index can be used to evaluate the dietary quality of a single meal (31).

#### Parent feeding styles

Parent feeding style was assessed using the Caregiver's Feeding Styles Questionnaire (3). This questionnaire measures feeding styles within a general parenting style framework. With the use of a 5-point scale (from 1 = never to 5 = always), parents report on how often they use 12 parent-centered compared with 7 child-centered feeding directives. Parents are then classified into 4 feeding styles on the basis of high and low scores of 2 dimensions of parents’ demandingness of and responsiveness to their child. Scoring procedures are described elsewhere (3–5). Reliability and validity of the measure have been established across multiple studies (3–5), including an observational study of similar design and study population as the present study (4).

#### Anthropometric measures

Trained research staff measured the height (Seca 213) and weight (Befour PS6600) of children and parents in light clothing without shoes to the nearest 0.1 cm and 0.1 kg, respectively. Children’s age- and sex-specific BMI percentile and standardized BMI (zBMI) were computed from the revised 2000 growth charts and classified according to the weight categories proposed by the CDC (32).

#### Child and parent characteristics

Parents and their children were classified as African American or Hispanic on the basis of self-identification. Parents also identified their educational attainment and their marital status, as well as their own and, if applicable, their spouse's employment status.

### Data analysis

Data analysis was conducted with the use of SPSS version 25. Missing data were handled using list-wise deletion. Parents were categorized into 4 feeding style groups by conducting a cross-classification of high and low median scores on the parent dimensions of demandingness and responsiveness from the Caregiver's Feeding Styles Questionnaire. The child HEI scores were derived from averaging across the dietary data derived from 3 meal observations. Three meals were observed for 89% of the families, 2 meals for 10% of the families, and 1 meal for 1% of the families. Chi-square tests and ANOVAs were conducted to assess differences in child, parent, and meal characteristics by feeding style. Differences in child HEI consumed scores between child, parent, and meal characteristics were examined using *t* tests and ANOVAs. Pearson's correlation was conducted to determine the correlation between HEI served and consumed scores, and exploratory paired-samples *t* tests were used to compare HEI served and consumed scores among child, parent, and meal characteristics. Three ANOVA models were conducted to compare differences in child HEI consumed score across feeding styles. The first was unadjusted, the second adjusted for HEI served score, and the third adjusted for child zBMI, parent ethnicity, parent education, parent employment, parent marital status, the number of people at the meal, the person serving the child, meal representativeness, and child HEI served score. A post hoc least-significant difference test was completed to determine which feeding styles differed from one another.

## RESULTS

Of the 275 families who consented to participate in this study, dietary information from observed dinner meals was available for 145 parent-child dyads. Only 131 dyads were included in this analysis due to incomplete feeding style (*n* = 6), and demographic (*n* = 8) data. Half of the preschoolers were male and 62% were Hispanic, and they had a mean ± SD age of 4.5 ± 0.6 y. Further descriptive characteristics of the sample are reported in **Table 1**. Chi-square and ANOVA testing indicated no significant differences in characteristics by feeding style. Independent *t* tests analyzing child HEI consumed scores by child, parent, and meal characteristics indicated a significantly higher mean HEI score consumed among Hispanic than among African-American families [*t* = 3.2, *P* = 0.002]. No other significant differences were found in HEI consumed scores within any of the child, parent, or meal characteristics.

**TABLE 1:** Characteristics of the study population: full analytic sample and by caregiver feeding style^1^

	Overall	Authoritative	Authoritarian	Indulgent	Uninvolved
Overall sample, *n* (%)	131 (100)	27 (21)	41 (31)	37 (28)	26 (20)
Child characteristics					
Age, y	4.5 ± 0.6^2^	4.6 ± 0.625	4.5 ± 0.6	4.5 ± 0.7	4.4 ± 0.7
Sex, *n* (%)					
Female	66 (50)	15 (56)	23 (56)	18 (49)	10 (38)
Male	65 (50)	12 (44)	18 (44)	19 (51)	16 (62)
zBMI	0.8 ± 1.1	0.6 ± 1.2	0.7 ± 1.0	0.9 ± 1.2	1.2 ± 1.1
Weight category, *n* (%)					
Underweight	3 (2)	1 (4)	0 (0)	2 (5)	0 (0)
Normal weight	74 (56)	16 (59)	29 (71)	15 (41)	14 (54)
Overweight	27 (21)	5 (19)	5 (12)	12 (32)	5 (19)
Obese	27 (21)	5 (19)	7 (17)	8 (22)	7 (27)
Parent characteristics, *n* (%)					
Race/ethnicity					
Hispanic	81 (62)	22 (81)	26 (63)	18 (49)	15 (58)
African American	50 (38)	5 (19)	15 (37)	19 (51)	11 (42)
Education					
Some college or more	57 (44)	13 (48)	16 (39)	17 (46)	11 (42)
High school diploma/GED	34 (26)	5 (19)	13 (32)	10 (27)	6 (23)
Some high school or less	40 (31)	9 (33)	12 (29)	10 (27)	9 (35)
Employment status					
Both caregiver and spouse employed	33 (25)	8 (30)	13 (32)	9 (24)	3 (12)
Either caregiver or spouse employed	72 (55)	15 (56)	19 (46)	19 (51)	19 (73)
Neither caregiver nor spouse employed	26 (20)	4 (15)	9 (22)	9 (24)	4 (15)
Marital status					
Married/cohabitating	64 (49)	14 (52)	24 (59)	14 (38)	14 (54)
Single	67 (51)	13 (48)	17 (41)	23 (62)	12 (46)
Meal characteristics					
Number of people at the meal	2.1 ± 1.1	2.0 ± 1.2	2.0 ± 1.0	2.5 ± 1.1	2.1 ± 1.3
Person serving child, *n* (%)					
Parent serves child	116 (89)	23 (85)	36 (88)	33 (89)	24 (92)
Child serves him/herself	15 (11)	4 (15)	5 (12)	4 (11)	2 (8)
Meal representativeness, *n* (%)					
Usual dinner	92 (70)	20 (74)	25 (61)	26 (70)	21 (81)
Not a usual dinner	39 (30)	7 (26)	16 (39)	11 (30)	5 (19)

1 No significant differences in characteristics by feeding styles were indicated by ANOVA or chi-square test. GED, general equivalency diploma; zBMI, standardized BMI.

2 Mean ± SD (all such values).

**Table 2** shows the results of paired-samples *t* tests between mean child HEI scores served and consumed. Overall, the HEI score of what children were served and what they consumed was highly correlated (*r* = 0.8, *P* < 0.001). However, significant differences between served and consumed scores existed among children whose parents had authoritarian or indulgent feeding styles compared with the authoritative feeding style (*P* = 0.008 and 0.044, respectively).

**TABLE 2:** Child HEI score of dinner meal served and consumed by characteristics^1^

	HEI dinner score	
	Served	Consumed
Overall	44.2 ± 8.4	43.4 ± 7.0
Parental feeding style		
Authoritative	47.5 ± 9.9	47.6 ± 6.8
Authoritarian	43.6 ± 8.1	41.5 ± 6.2^2^
Indulgent	44.7 ± 7.0	43.6 ± 6.4^3^
Uninvolved	41.2 ± 8.1	41.7 ± 7.8
Child characteristics		
Sex		
Female	43.0 ± 8.2	42.8 ± 6.5
Male	45.5 ± 8.5	43.9 ± 7.6^2^
Weight category		
Underweight	45.0 ± 5.0	45.9 ± 3.7
Normal weight	44.3 ± 1.0	43.3 ± 0.9
Overweight	43.0 ± 1.6	43.8 ± 1.3
Obese	45.3 ± 1.5	42.9 ± 1.4^3^
Parent characteristics		
Race/ethnicity		
Hispanic	46.0 ± 9.2^4^	44.9 ± 7.4^4^
African American	41.4 ± 6.0	41.0 ± 5.8
Education		
Some college or more	44.2 ± 8.0	43.6 ± 7.1
High school diploma/GED	43.0 ± 8.8	42.3 ± 6.6
Some high school or less	45.4 ± 8.7	44.03 ± 7.4
Employment status		
Both caregiver and spouse employed	46.3 ± 8.31	44.7 ± 6.6
Either caregiver or spouse employed	43.4 ± 8.81	43.1 ± 7.6
Neither caregiver nor spouse employed	44.0 ± 7.1	42.5 ± 5.9^3^
Marital status		
Married/cohabitating	45.0 ± 9.3	44.3 ± 6.8
Single	43.5 ± 7.5	42.5 ± 7.3^3^
Meal characteristics		
Person serving child		
Parent serves child	44.4 ± 0.8	43.6 ± 0.7
Child serves themselves	42.7 ± 1.5	41.7 ± 1.7
Meal representativeness		
Usual dinner	44.2 ± 0.9	43.4 ± 0.7
Not a usual dinner	44.4 ± 1.3	43.4 ± 1.1

1 Values are means ± SDs; *n* = 131. GED, general equivalency diploma; HEI, Healthy Eating Index.

2, 3 Different from HEI dinner score served (paired-samples *t* test; differences assessed horizontally within a row): ^2^*P* < 0.01, ^3^*P* < 0.05.

4 Different across levels of the characteristic, *P* < 0.05 (paired-samples *t* test indicated the HEI dinner score to be significantly different; assessed vertically within a column).

**Table 3** shows the unadjusted and adjusted means of child HEI consumed scores. All 3 ANOVA models showed a significant difference in child HEI consumed score across feeding styles [unadjusted *F*_(3, 116)_ = 5.1, *P* = 0.002, _η_^2^ = 0.1; partially adjusted *F*_(3, 116) _= 3.9, *P* = 0.011, _η_^2^ = 0.1; fully adjusted *F*_(3, 116) _= 3.7, *P* = 0.015, _η_^2^ = 0.1]. Post hoc tests using least-significant differences showed a significantly higher HEI consumed score among the authoritative feeding style compared with the authoritarian, indulgent, and uninvolved feeding styles in the unadjusted model (*P* < 0.001, *P* = 0.021, and *P* = 0.002, respectively). The higher HEI-consumed score among the authoritative compared with the uninvolved feeding style was no longer significant in the model adjusting for HEI-served score (*P* = 0.118), and only the difference between authoritative and authoritarian feeding styles remained significant in the fully adjusted model (*P* = 0.001).

**TABLE 3:** Adjusted and unadjusted mean child HEI scores of dinner meal consumed by feeding style^1^

	Child HEI consumed score					
	Unadjusted model		Partially adjusted model^2^		Fully adjusted model^3^	
Caregiver feeding style	Mean ± SD	*P*	Mean ± SEE	*P*	Mean ± SEE	*P*
Authoritative	47.6 ± 6.8	–	45.5 ± 0.8	–	45.5 ± 0.9	–
Authoritarian	41.5 ± 6.2	<0.001	41.9 ± 0.7	0.001	41.9 ± 0.7	0.001
Indulgent	43.6 ± 6.4	0.021	43.3 ± 0.7	0.042	43.2 ± 0.7	0.059
Uninvolved	41.7 ± 7.8	0.002	43.6 ± 0.8	0.118	43.7 ± 0.9	0.161

1 *P* values were determined by using least-significant differences pairwise comparison. HEI, Healthy Eating Index; zBMI, standardized BMI.

2 Adjusted for child HEI served score.

3 Adjusted for child zBMI, parent ethnicity, parent education, parent employment, parent marital status, the number of people at the meal, the person serving the child, meal representativeness, and child HEI served score.

## DISCUSSION

This study examined the relation between parent feeding style and overall preschooler dietary quality at dinner among a sample of low-income Hispanic and African-American families. Consistent with our hypothesis, the authoritative feeding style (high demandingness and high responsiveness) was associated with significantly higher child dietary quality than the authoritarian feeding style (high demandingness and low responsiveness). The superior dietary quality found here for the authoritative compared with the authoritarian feeding style is consistent with child dietary outcomes from research with general parenting styles, which found fruit and vegetable consumption to be better among children with parents engaging in an authoritative feeding style (23). In contrast to our hypothesis, in the adjusted model, no significant differences in child dinner dietary quality were seen between the authoritative feeding style and either the indulgent or uninvolved feeding styles. This is not consistent with previous studies on feeding style and dietary quality, which found permissive feeding styles to be associated with higher child consumption of low-nutrient-dense foods and poor fruit and vegetable consumption (8, 9). This is likely due to the great number of covariables in the main model and the overall sample size. In the unadjusted model, the authoritative feeding style was associated with significantly higher dietary quality compared with all other feeding styles. Overall, the main findings of this study indicate that in low-income minority families, a child's highest dietary quality is likely to be achieved when parents not only set appropriate guidelines for eating but are also responsive to their child's eating preferences and behaviors.

Although most dietary studies rely on self-report, one strength of this study was its ability to objectively measure participants’ diet through detailed menu information, digital photography, and food-waste measurement. The dietary component in this study was unique because it includes the objective measurement of both food served and food consumed. Having both measurements enabled exploratory investigation into the differences in the quality of food eaten compared with that served to the children. Although consumption is ultimately limited by the food available, the child's choice of which foods and how much to consume of the meal served to him or her can either increase or decrease dietary quality.

The findings of this study are consistent with our exploratory hypothesis that the overall dietary quality of food served would be higher than that of the food consumed. Although this difference was not significant among the overall sample, the dietary quality of the meal served was significantly higher than the dietary quality of the meal consumed among children whose parents have either an authoritarian or indulgent feeding style. These feeding styles have both been associated with unhealthy dietary intake (8, 9). Parents with an indulgent feeding style are likely to be responsive to a child's mood during the meal but are unlikely to make demands on or offer guidance about a child's intake once the food is served. It is thought that this lack of guidance contributes to poor child dietary quality (8).

Conversely, parents with an authoritarian feeding style are likely to make demands of their child during dinner and not be responsive to their child's preferences, emotions, or individual needs. However, it is possible for the high level of demandingness exhibited by authoritarian feeders to consist of demands unrelated to overall dietary quality. For example, a parent could make disciplinary demands on a child during dinner (e.g., sit up straight, use your napkin). It is also possible for the demands made by parents with an authoritarian feeding style to be inconsistent with healthy choices. The parent feeding style construct does not specifically consider parents’ nutrition knowledge or values with regard to healthful food, but only assesses parents’ level of demandingness on and responsiveness to their children during eating episodes. The significant difference between the dietary quality served and consumed indicates that parents’ influence during a meal is important and necessitates more investigation into how specific goal-oriented parent feeding practices during the dinner meal may relate to overall dietary quality. This may be especially pertinent given that the dietary quality of the meal served was significantly higher than that consumed among children with obesity. The lack of statistical difference in zBMI across feeding styles seen in this sample (*n* = 131) could be due to insufficient power because a significant 3-way interaction between parent feeding style, ethnicity, and sex on child zBMI was found in the full sample (*n* = 177) (4).

Given that many Head Start preschool children eat breakfast, lunch, and 2 snacks at the Head Start center, this study captured the dietary quality of the meal most reflective of parents’ influence on their child's dietary quality: the dinner meal. Furthermore, the multiple-observation method used in this study offers insight into the family's dinner patterns. However, HEI scores for one meal may not be reflective of the dietary quality of an entire day's intake. The overall mean ± SD HEI score of the dinner meal consumed in the present study was lower (43.4 ± 7.0) than in previous studies that used dietary recall data from the 2012 NHANES, which reported an average HEI-2010 score of 52 for children aged 4–8 y (33) and 55 for children aged 2–5 y (34). Some food components are more likely to be consumed at various times of the day than are others, and this may differ by culture (35). For example, in a typical American diet, fruit may be more frequently consumed in the morning than in the evening meal. In this instance, a low fruit component score for dinner does not necessarily indicate that fruit is lacking from a person's diet. Because the majority of the sample in the present study was Hispanic (62%), it is unlikely that the low (relative to other preschool-aged children) dietary quality scores observed in this study were due to ethnic differences in eating patterns. Indicators of dietary quality—for example, vegetable intake—have consistently been found to be higher among Hispanic individuals than African-American or White individuals (36–38). Although a good strategy to ensure that all food components are regularly consumed, each food component does not need to be consumed at each meal for adequate overall dietary quality.

Some limitations to this study are acknowledged. As mentioned above, only the dinner meals were observed among various meals consumed by children during the day. Other limitations include the use of direct observation, which may alter family behaviors, including the food served by the parent and the food consumed by the child. In addition, the focus on only Head Start African-American and Hispanic families reduces the generalizability of these findings to low-income Head Start families from 2 ethnic groups. Last, due to the cross-sectional design of this study, no inferences with regard to causation can be made.

A nutritious diet is necessary for proper growth and development (11) and dietary consumption habits established during childhood are likely to continue being practiced as an adult (13). The results of this study suggest that, with regard to child dietary quality, an authoritative feeding style may be preferable to an authoritarian feeding style among low-income minority families with preschool-aged children. Further research to better understand feeding practices that contribute to authoritative feeding, especially those occurring during the dinner meal, is warranted to gain a better understanding for how parent feeding styles can be shifted toward the authoritative feeding style. From this information, parent feeding interventions can be designed to improve the dietary quality and, consequently, the health of children.

## Supplementary Material

Supplement Files

## Notes

Supported by funds from the USDA, grant 2006-55215-16695. This work is a publication of the United States Department of Agriculture [USDA–Agricultural Research Service (-ARS)] Children's Nutrition Research Center, Department of Pediatrics, Baylor College of Medicine, Houston, Texas, and has been funded in part with federal funds from the USDA-ARS under cooperative agreement 58-6250-0-008, and in part by Kraft Foods, Inc.

Supplemental Figure 1 is available from the “Supplementary data” link in the online posting of the article and from the same link in the online table of contents at https://academic.oup.com/ajcn/.
