# Supplementary material for: Authoritative parent feeding style is associated with better child dietary quality at dinner among low-income minority families
Source: Am J Clin Nutr. 2018 Aug 30;108(4):730–6. doi: 10.1093/ajcn/nqy142 (PMC6186208; doi:10.1093/ajcn/nqy142)
Supplement: Supplement Files [file nqy142_supplemental_table_figure.zip › Supplemental Figure.docx]

**Supplemental Figure 1: Participant Flow Diagram**

6 missing feeding style data

8 missing demographic data

275 Consented

145 Observed at Dinner

131 Analyzed

~2500 Eligible
